# Does Elevated [CO_2_] Only Increase Root Growth in the Topsoil? A FACE Study with Lentil in a Semi-Arid Environment

**DOI:** 10.3390/plants10040612

**Published:** 2021-03-24

**Authors:** Maryse Bourgault, Sabine Tausz-Posch, Mark Greenwood, Markus Löw, Samuel Henty, Roger D. Armstrong, Garry L. O’Leary, Glenn J. Fitzgerald, Michael Tausz

**Affiliations:** 1 51 Campus Drive, College of Agriculture, University of Saskatchewan, Saskatoon, SK S7N 5A8, Canada; 2 Northern Agricultural Research Center, Montana State University, 3710 Assinniboine Road, Havre, MT 59501, USA; 3 Faculty of Veterinary and Agricultural Sciences, The University of Melbourne, 4 Water Street, Creswick, VIC 3363, Australia; llow@swin.edu.au (M.L.); sam.henty@agriculture.vic.gov.au (S.H.); 4 Department of Agriculture, Science and the Environment, CQ University Australia, 114-190 Yaamba Road, Norman Gardens, QLD 4701, Australia; s.tausz-posch@cqu.edu.au (S.T.-P.); m.tausz@cqu.edu.au (M.T.); 5 Department of Mathematical Sciences, Montana State University, Bozeman, MT 59717, USA; greenwood@montana.edu; 6 Agriculture Victoria, The University of Melbourne Campus, Parkville, VIC 3053, Australia; 7 Agriculture Victoria, Grains Innovation Park, 110 Natimuk Road, Horsham, VIC 3401, Australia; roger.armstrong@agriculture.vic.gov.au (R.D.A.); garry.oleary@agriculture.vic.gov.au (G.L.O.); glenn.fitzgerald@agriculture.vic.gov.au (G.J.F.)

**Keywords:** *Lens culinaris*, climate change adaptation, root development, root depth distribution

## Abstract

Atmospheric carbon dioxide concentrations [CO_2_] are increasing steadily. Some reports have shown that root growth in grain crops is mostly stimulated in the topsoil rather than evenly throughout the soil profile by e[CO_2_], which is not optimal for crops grown in semi-arid environments with strong reliance on stored water. An experiment was conducted during the 2014 and 2015 growing seasons with two lentil (*Lens culinaris*) genotypes grown under Free Air CO_2_ Enrichment (FACE) in which root growth was observed non-destructively with mini-rhizotrons approximately every 2–3 weeks. Root growth was not always statistically increased by e[CO_2_] and not consistently between depths and genotypes. In 2014, root growth in the top 15 cm of the soil profile (topsoil) was indeed increased by e[CO_2_], but increases at lower depths (30–45 cm) later in the season were greater than in the topsoil. In 2015, e[CO_2_] only increased root length in the topsoil for one genotype, potentially reflecting the lack of plant available soil water between 30–60 cm until recharged by irrigation during grain filling. Our limited data to compare responses to e[CO_2_] showed that root length increases in the topsoil were correlated with a lower yield response to e[CO_2_]. The increase in yield response was rather correlated with increases in root growth below 30 cm depth.

## 1. Introduction

Atmospheric carbon dioxide concentrations [CO_2_] are increasing steadily, rising from approximately 280 ppm before the industrial age to approximately 413 ppm today (December 2020; www.co2.earth (accessed on 25 January 2021)). In the absence of changes in temperatures and precipitation patterns, elevated [CO_2_] (e[CO_2_]) in C3 crops has been associated with higher photosynthetic rates and lower stomatal conductance, leading to greater biomass and grain yield, as well as greater transpiration efficiency [1,2]. While we have ample data on the effects of e[CO_2_] on above-ground biomass albeit not always consistent, data on the effects of e[CO_2_] on below-ground processes are more limited, especially in agricultural systems [3,4]. Considering the intimate relationship between resource acquisition and growth, understanding the above-ground response to e[CO_2_] and its variability might be better achieved through understanding the response of root systems [3].

Roots are the primary interface of water and nutrient acquisition from the soil, and yet are the most under-researched organ due to the difficulty in observing them in situ. A better understanding of root morphological and physiological traits is the new frontier in understanding adaptation to dryland cropping [5]. While the prevailing view has generally been to assume a large root system would prevent against the negative impacts of drought by providing better access to soil water, Passioura [6] suggested several decades ago that the extraction of too much water too early and excessive carbon allocation to roots under such scenario might be counterproductive. Palta et al. [7] and Lynch [8] recently expanded on this idea by suggesting the optimal size of a crop root system would depend on the typical pattern of water availability during the season [7] and input into the cropping system, with a parsimonious root system likely to be more efficient, especially in high-input systems [8].

A meta-analysis showed that e[CO_2_] generally increases both shoot and root biomass [4]. However, some reports have shown that root growth of grain crops is preferentially increased in the topsoil rather than evenly through the soil profile [9,10,11]. For example, Qiao et al. [10] showed that although root length in the surface layers was increased 35 to 45% in wheat, root length at depth was reduced, leading to lower soil water extraction below 1 m with e[CO_2_]. This change, if consistent, might not be beneficial for crops growing in semi-arid environments, especially if grown on stored water, and could lead to a lack of response to e[CO_2_], or even potentially, lower grain yields.

Lentil is a cool-season, indeterminate crop whose global production has more than quadrupled since the 1960s. Expansion into new areas such as temperate regions of Australia, the Canadian Prairies and the Northern Great Plains of the United States has led to a global production of over 6.3 million metric tons in 2018 [12]. In Australia, it is grown as a winter crop and is subject to low temperatures during the vegetative stage followed by high temperature stress by flowering and pod filling, often combined with water stress. Bourgault et al. [13] showed that the grain yield response of lentil to e[CO_2_] was considerable (average = 42%) but also highly variable (18–138%).

Therefore, the objective of this study was to investigate the effect of e[CO_2_] on root growth and soil water extraction in lentil compared to growth at ambient [CO_2_], with a special emphasis on the distribution of roots down the soil profile. Our hypothesis was that there might be disproportional root growth in the surface layers, leading to a lower response to e[CO_2_] in semi-arid areas. This is the first report of season-long non-destructive root growth observations in lentils grown under Free Air CO_2_ Enrichment (FACE).

## 2. Results

**Figure 1:**
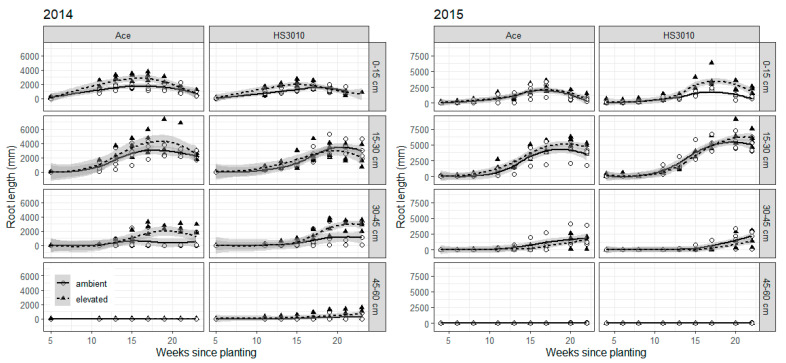
Root length captured by mini-rhizotrons for lentil by [CO_2_], cultivar and depth in the Australian Grains Free Air CO_2_ Enrichment in 2014/2015. A [CO_2_] by time by depth interaction was significant in 2014 and a [CO_2_] by cultivar by time by depth interaction was significant in 2015. Flowering occurred at week 17 in both years. Round white circles represent individual sub-plots in ambient main plot and black triangles individual sub-plots in elevated [CO_2_] main plots. Grayed areas are the calculated confidence interval for treatment means as calculated by the ggplot2 R package. Symbols may be drawn on top of one another.

Root length was not increased by e[CO_2_] equally across all depths. In 2014, e[CO_2_] increased root length at depths 0–15 cm and 30–45 cm (Figure 1). Root length increases are particularly evident prior to flowering for depth 0–15 cm with an average increase of 65% between weeks 11 to 15 in the cultivar PBA Ace, and 72% for the cultivar HS3010 over the same period. At depth 30–45 cm, e[CO_2_] increased root length by 266% and 170% for PBA Ace and HS3010, respectively, during grain filling (week 21) although the absolute value of the root increase was greater in HS3010 than PBA Ace. In 2015, e[CO_2_] significantly increased root length at depth 0–15 cm for the genotype HS3010 but at 15–30 cm for the cultivar PBA Ace (Figure 1). At flowering (week 17), root length under e[CO_2_] was increased 90% in the topsoil for HS3010. By contrast, root length in Ace was increased 15% at depth 15–30 cm at this same time point (Figure 1).

**Figure 2:**
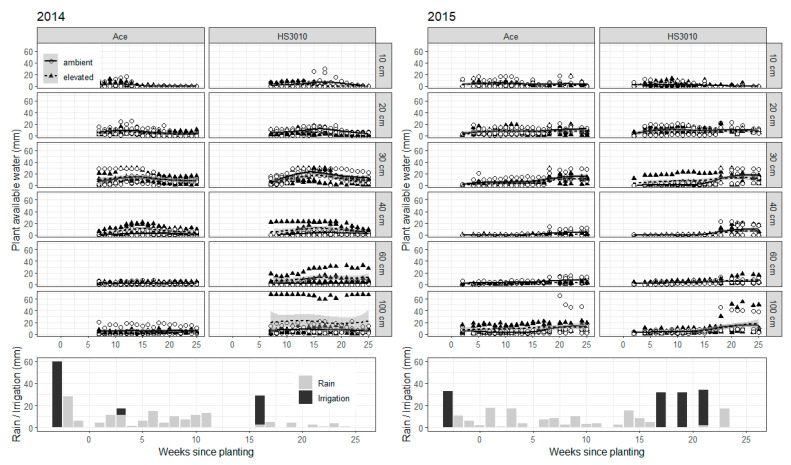
Plant available water for lentil by [CO_2_] and depth in the Australian Grains Free Air CO_2_ Enrichment in 2014–2015 with rainfall and irrigation events during the season. In both years, a [CO_2_] by cultivar by time by depth interaction was significant in 2015. Planting occurred at week 0, flowering at week 17, and harvest at week 25 in both years. Round white circles represent individual sub-plots in ambient main plot, and black triangles individual sub-plots in elevated [CO_2_] main plots. Grayed areas are the calculated confidence interval for treatment means as calculated by the ggplot2 R package. Symbols may be drawn on top of one another.

There were significant 4-way interactions in both 2014 and 2015 for the plant available water data, although this was possibly due to some tubes starting with higher values at the beginning of the season (Figure 2). Pre-flowering and post-flowering water use showed cultivar differences in 2014, with the cultivar HS3010 showing less water use pre-anthesis, but more post-anthesis (Table 1). These cultivar differences were not detected in 2015, and no [CO_2_] treatment differences were obvious from these data in either year. Both seasons were quite dry, but the timing of soil water availability also differed with more soil water available pre-flowering in 2014, whereas in 2015 more soil water was available between flowering and grain filling compared to 2014, due mainly to emergency irrigation events (Table 1).

**Figure 3:**
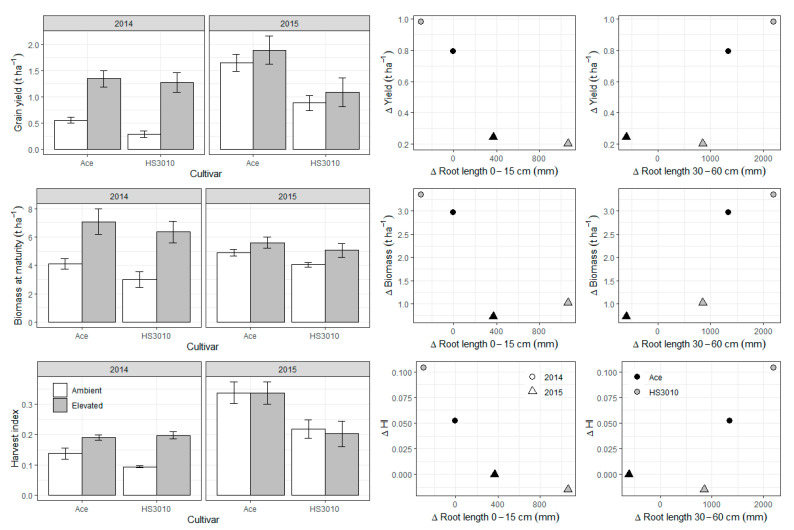
Grain yield, biomass at maturity and harvest index means over CO_2_ treatments by years and cultivars in lentil and selected relationships in absolute differences between e[CO_2_] and a[CO_2_] in these parameters with increases in root length at 0–15 cm and 30–60 cm at grain fill. Barplots are a re-analysis of the data presented in Bourgault et al. [13].

**Figure 4:**
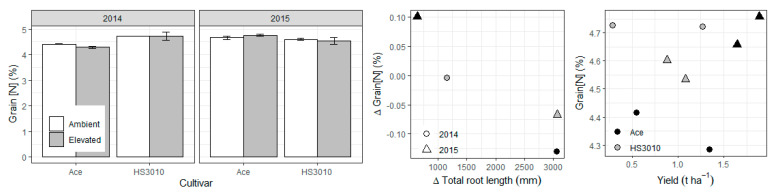
Grain [N] means over CO_2_ treatments by years and cultivars in lentil and selected relationships with increases in total root length at 0-15 cm and grain yield. Barplot is a re-analysis of the data presented in Bourgault et al. [13].

Since AGFACE rings were not paired, there is limited replication to directly compare responses. However, differences in root length between e[CO_2_] and a[CO_2_] were associated with different [CO_2_] responses in grain yield, biomass at maturity, and harvest index (HI) depending on the depth where the increase in root growth occurred (Figure 3). There was marginally significant exponential decay relationships between increases in root length in the topsoil at the grain filling stage due to e[CO_2_] and responses in grain yield (*p* = 0.09) and HI (*p* = 0.08), suggesting a trade-off in carbon (C) allocation between roots and above-ground biomass. By contrast, there were positive but non-significant linear relationships between increases in root length below 30 cm (again at the grain filling stage) and responses in these same parameters (*p* = 0.14, *p* = 0.12 and *p* = 0.20). Relationships between root length and grain [N] were less clear, but there was a marginally significant (*p* = 0.08) negative linear relationship between differences in total root length at grain filling and grain [N], so that increases in root length over the soil profile with e[CO_2_] were associated with decreases in grain [N] with e[CO_2_] (Figure 4).

## 3. Discussion

The large increase in root length observed at the 30–45 cm depth in both genotypes in 2014 suggests that e[CO_2_] did not, by itself, disproportionally increase root development in the top soil layers. Rather, the response in root growth due to e[CO_2_] appeared to be strongly related to the availability of soil water in the soil profile. In fact, Nie et al. [4] showed that the proportion of roots in the topsoil in field experiments was generally reduced by e[CO_2_] by approximately 8%. However, they also noted that out of the 17 studies that report this information, the 5 agricultural studies were clear outliers compared to grassland and forest studies and showed an increase of approximately 4%. It is worth noting that agricultural studies tend to be irrigated, often with frequent irrigation events, which may or may not fill the entire profile. For example, Saha et al. [11] applied “moderate irrigation (20 mm) […] whenever required”, and Wechsung et al. [9] used a subsurface (0.18–0.25 m) drip irrigation system with 50% replacement of potential evapotranspiration for their drought treatment. It is possible that soil water was more available at the surface, or near the drip line, and that root increases seen with e[CO_2_] would be partly explained by soil water availability differences within the profile rather than depth per se.

Increases in root length in the top layer of the soil profile appeared to be related to a smaller response in grain yield, and characterized by an exponential decay (Figure 3). Although we have limited data points to observe this relationship from rings not being paired, this suggests that root length increases in the topsoil due to e[CO_2_] may be a maladaptive response, at least in this environment. This is consistent with results from Benlloch-Gonzalez et al. [14] who demonstrated a lack of above-ground biomass response in a high vigor wheat line that showed greater root growth in the top layers and less root growth at depth under e[CO_2_]. It suggests that allocation of the additional C to root growth in the top layer rather than to above-ground biomass is limiting the response to e[CO_2_]; presumably greater leaf area at flowering is more important to continue to capture the growth benefits from e[CO_2_] than additional access to water (if any) or nutrients in surface layers of the soil.

In contrast, root length increases at 30–60 cm due to e[CO_2_] were related to an increasing yield and biomass response to e[CO_2_], not just linearly, but exponentially. Again, more data are needed to confirm the shape of the relationship, if any, between above-ground parameter responses and increases in root growth at depth in agricultural crops. Other studies have reported comparisons that are consistent with our results: Uddin et al. [15], in the SoilFACE large soil core facility that is also part of AGFACE, showed that the canola genotype with the greater stimulation of roots at depth (41–80 cm) also showed the greater yield response to e[CO_2_]. They also showed that in wheat, in the same environment as the current study, root growth at 45–60 cm depth was correlated with water use, biomass and yield, but root growth at 0–15 cm was not [16]. Roots deeper in the soil profile can extract water during grain filling and have been shown to be particularly useful for yield formation [17,18]. Manschadi et al. [17] for example showed that “each additional millimeter of water extracted [at depth] during grain filling generated an extra 55 kg ha^−1^ of [wheat] grain yield”. However, the difference in soil water availability at the 30 and 40 cm depths (Figure 2) between the two years points to how the development of root growth at depth also depends on soil water availability in shallower depths. This raises concerns in semi-arid cropping systems that rely partially or fully on stored soil water, a dry soil layer due to continuous cropping and/or intermittent drought would likely reduce or eliminate crop responses to e[CO_2_].

The greater yield response to e[CO_2_] seen with less root development in the surface layers and more root development at depth echoes findings from retrospective studies looking at root growth, water use and water use efficiency in breeding populations of both maize [19] and wheat [20] for semi-arid environments. Bolanos et al. [19] showed that yield improvement in maize was associated with 33% less root mass at the 0–50 cm layer, but they did not find any differences in plant water status between old and new cultivars. Pask and Reynolds [20] similarly showed that over time the proportion of root biomass shifted towards deeper soil layers, which improved water use, but not water use efficiency. Selection for such a trait therefore appears useful in itself but might be particularly necessary to take advantage of the increasing atmospheric [CO_2_].

We expected that increased root growth under e[CO_2_] would have led to faster root exploration into deeper soil layers, with roots detected at depths 30–45 or 45–60 cm in e[CO_2_] plots before ambient plots. For example, Chaudhuri et al. [21] investigating root growth with winter wheat in a rhizotron facility showed that roots reached the bottom of the pots faster under e[CO_2_] than under a[CO_2_]. We could not however detect such differences in our experiment with scans being taken every second week.

Grain [N] is often decreased under future high CO_2_, negatively affecting the nutritional and economic value of crops [22]. While results on cereals show universal decreases in grain [N], results on pulses are inconclusive. Under ideal growing conditions pulses may balance the increase in carbon source by increasing symbiotic N2 fixation, thus avoiding imbalances in the C:N ratio, but this benefit has proven elusive under more challenging growing conditions, especially under drought and heat [23,24]. This is consistent with the findings of the current study where grain [N] was generally maintained under e[CO_2_], but was significantly decreased in the drier 2014 season in cultivar Ace (*p* = 0.0279; Figure 4), indicating that the source of N (whether from N_2_ fixation or from soil uptake) feeding into the grain can be affected by the amount and timing of water availability [25,26].

The relationship between the difference (in absolute values) in grain [N] due to e[CO_2_] and the difference in total root length was marginally significant (*p* = 0.08; Figure 4). The negative relationship indicates that increases in root length over the soil profile with e[CO_2_] were associated with decreases in grain [N]. This negative relationship coincides with the positive relationship between increases in root length and increases in yield (Figure 3), although the well-known negative relationship between grain yield and grain [N] (or grain protein more generally) is not significant in this case (Figure 4; *p* = 0.70). It is noteworthy that Bahrami et al. [27] did not find a relationship between grain protein and root traits in wheat and the authors suggested that root growth stimulation (or any changes in specific root uptake activity) under e[CO_2_] were insufficient to alleviate the negative effect of e[CO_2_] on wheat grain protein.

Root length and soil water data have inherently high variability, especially when measured under field conditions and this limits the ability to distinguish clear treatment differences. Before the beginning of this experiment, we were concerned that the images obtained with mini-rhizotrons might underestimate the root length in the surface layer and overestimate root length at depth, especially if roots took the path of least resistance and followed the outer wall of the tube down the profile. While we did not have the resources to take root biomass samples in addition to root images, it appears that the mini-rhizotrons did underestimate root length at the surface (based on published root mass distribution in various crops [21,28,29], but sufficient root growth was detected that the bias should be consistent between [CO_2_] and cultivar treatments. We also found that while roots would follow the tube down for some length, they did not generally go all the way down, but often turned back into the soil. Many images at depth had no roots visible at all, which might partly explain the lack of significance for some apparent differences between cultivars. For example, we might have expected a cultivar by depth interaction in 2014 due to the growth of roots at the 45–60 cm depth in HS3010 and lack of root growth in the cultivar Ace at this same depth, but the *p*-value for this interaction was 0.547. Some images were also lost in 2014 due to poor image quality arising from soil cracks (in this cracking clay soil) interfering with image capture. This problem was corrected in 2015 by covering the top of the tube with a carboard box. In a different experiment (at a different site), the coefficient of variation was around 40–48% for depths 0–15, 15–30 and 30–45 cm, and up to 87% for depth 45–60 cm for the check cultivar (Bourgault et al., unpublished). Ohashi et al. [30] suggested using at least 3 replications; we would rather suggest using 5 replications or more, and carefully evaluating the level of differences the researchers would like to be able to detect statistically.

Similarly, PAW showed some differences between tubes that remained consistent throughout the season, which suggests there might have been micro-environment differences between the tubes that were not captured in the depth-wide calibration (Figure 2). Freebairn and Ghahramani [31] showed that PAW is generally accurate in the ±50 mm range, and although errors are expected to go down proportionally as the absolute number gets lower, small [CO_2_] treatment differences in water use (in the range of 2–10 mm) might be too challenging to detect considering typical soil variability.

## 4. Materials and Methods

### 4.1. Site Location and Climate

The experiment was conducted in 2014 and 2015 at the Australian Grains Free Air CO_2_ Enrichment (AGFACE) facility, near Horsham, VIC, Australia (36°45′07″ S, 142°06′52″ E, 127 m above sea level), on a Vertosol grey clay [32]. Long-term annual rainfall at this site is 435 mm (based on the 1981–2010 period), with approximately 320 mm falling during the winter growing season, i.e., from May to November inclusively [33]. However, 2014 and 2015 were considered very dry years, with only 221 and 214 mm of annual rainfall, respectively, and growing season rainfall (April to November), of 110 and 120 mm, respectively. Average maximum and minimum temperatures were 17.6 and 5.3 °C, respectively, during the growing season. In both years, warmer-than-average temperatures in October accelerated lentil development leading to early harvests for the region [34,35]. In addition, in 2015, a late frost on 1 October (−0.2 °C) was followed by a heat wave on October 4–6th (with maximum temperatures of 35–37 °C).

### 4.2. Site Management

Plots were planted on 12 May 2014 and 26 May 2015. Pre-emergence herbicides (simazine, trifluralin, isoxaflutole, and/or glyphosate) were applied to help manage weeds. Superphosphate was placed with the seed at sowing at a rate equivalent to 9 kg P ha^−1^ and 11 kg S per ha^−1^. Seeds were inoculated with a granular pea and lentil inoculant in 2014 (Nodulator, BASF Corporation, Research Triangle Park, NC, USA) and a peat-based inoculant in 2015 (NoduleN, New Edge Microbials Pty Ltd., Albury, NSW, Australia). No nitrogen fertilizer was applied. In 2014, the insecticide dimetoate was applied to control aphid, and the fungicide chlorothalonil was applied to prevent against ascochyta blight. Weeds were controlled by hand during the season. Pre-planting irrigation events were applied (60 mm in 2014 and 33 mm in 2015), as well as emergency irrigation events during the season to alleviate the most severe water stress: in 2014, 6 mm irrigation was applied on 16 June to help emergence, and another 26 mm irrigation was applied on 3–5 September; in 2015, three 32 mm irrigations were applied on 21–22 September, 8–9 October, and 4–6 October (Figure 2).

### 4.3. FACE Technology

In AGFACE, elevated [CO_2_] was achieved by injecting pure CO_2_ into the air: stainless steel tubes were positioned about 50 cm above the canopy, and depending on wind speed and direction, CO_2_ was released upwind so as to be carried across the ring. The target [CO_2_] for the elevated treatment was 550 mmol mol^−1^ air. Concentrations were maintained within 90% of this target (i.e., 500–600 mmol mol^−1^ air) 93–98% of the time [36]. More details about the exposure equipment are given in Mollah et al. [36].

### 4.4. Experimental Design

The experiment was a randomized complete block split-plot design with 4 blocks, each containing one ambient and one elevated [CO_2_] octagonal ring, as the main plots. Within the main plots, different genotypes were grown in subplots. In 2014, rings were 4 m in diameter with six subplots of 4 rows (0.244 m row spacing) by 2 m in length (total subplot size: 1.95 m^2^) containing six lentil cultivars but only two equipped with mini-rhizotrons. Randomization within the main plot was restricted to have these cultivars end to end in a middle row. In 2015, lentil subplots were included in 12-m diameter rings containing primarily wheat, with subplots of 6 rows (0.25 m row spacing) by 4 m in length (total subplot size: 6 m^2^). Randomization within the ring was also restricted to have lentil plots together end to end for ease of access to mini-rhizotron tubes. More details are available in Bourgault et al. [13,24].

### 4.5. Plant Material

The commercial cultivar PBA Ace and the breeding line 05H010L-07HS3010 (shortened to HS3010 hereafter) were selected for this study. Both lines are medium-sized red lentils with similar phenology. PBA Ace is a modern variety that performs well under a range of Australian conditions. By contrast, the breeding line HS3010 had previously showed large biomass accumulation in favorable growing conditions but showed a smaller harvest index than most commercial lines (M. Rodda, personal communication). Lentil is often thought of as source-limited, so these two contrasting lines were considered particularly interesting to examine in terms of the effect of e[CO_2_] on both above-ground and below-ground processes.

### 4.6. Root Growth Observations

Clear acrylic tubes 105.5 cm long by 7.5 cm diameter (i.e., mini-rhizotrons) were installed at a 45° angle within 7 days of planting. These were installed between center rows of experimental plots, but towards the front of the plot (within 50–70 cm) to limit traffic inside the plots. Every 2–3 weeks, root scans were taken with a cylindrical scanner (CI-600 In Situ Root Imager, CID Bioscience, Camas, WA, USA). Four images were taken per tube with depths approximating 0–15, 15–30, 30–45, and 45–60. The length of the tube out of the ground was recorded, and the angle measured after installation. Overall, the angle varied by 5°, and the length out of the ground varied by at most 8 cm, so that the maximum difference in depth for images was 2–5 cm. Images were later processed with the *RootSnap!* software (CID Bioscience, Camas, WA, USA) and the root length for each image recorded.

### 4.7. Soil Water Monitoring

Soil water was monitored with a PR2/6 profile probe (Delta-T Devices, Cambridge, UK), which outputs soil water at depths 10, 20, 30, 40, 60, and 100 cm. Access tubes were installed between center rows of the experimental plots but not in the same row as the mini-rhizotrons. They were also installed further inside the plots (1–2.5 m from the front, depending on the size of the plot). Measurements were taken on a weekly basis for the duration of the experiment, except early in the season in 2014 when there were delays in receiving and installing the profile probe and access tubes. A manual calibration was performed for each depth using the soil samples taken at the time of the tube installation and assessed gravimetrically in both years. Plant available water (PAW) was calculated for each depth from the volumetric water content using values for the wilting point and for field capacity (by O’Leary et al. [37]). These data are presented in Figure 2, along with rainfall and irrigation events. Pre-flowering and growing season water use were calculated by taking the difference in PAW across depths between the first and last PR2 measurements of the period (week 16 for pre-flowering, therefore not including flowering), or season and adding rainfall and irrigation events. Post-flowering water use was calculated from the difference between season-long and pre-flowering water use. This water use is an approximation of evapotranspiration as it includes water lost through evaporation but assumes no runoff or deep drainage. In 2014, there was a delay in getting the equipment, and these data are therefore potentially under-estimated.

### 4.8. Other Measurements

Biomass, grain yield and yield components were previously published in Bourgault et al. [13,24]. Briefly, destructive samples were collected at flowering (full bloom) and maturity stages according to the description of phenological stages in lentil by Erskine et al. [38]. Samples were separated into leaf, stem and flower tissues at flowering, and grain and straw (dead leaves and stems) at maturity. These tissues were ground and analyzed separately for nitrogen concentration by LECO Tru Mac Elemental Analyser (LECO Corporation, St. Joseph, MI, USA).

### 4.9. Statistics

The root length data, by nature, contained many values of zero, especially at the beginning of the season and at depth. In addition, root length over the season followed a typical growth curve, but the curvature varied by depth. As such, the structure of the data included non-constant variance as well as time- and depth-related curvatures. Accordingly, the root length data were transformed by taking the square root, and the time factor was considered as a numerical cubic polynomial factor based on week from planting. Each year was analyzed separately because the timing of sampling differed slightly between years and because environmental conditions differed between the two years. Fixed factors included [CO_2_], cultivar, time, depth, and all interactions between these four factors. A mixed model was used to account for the measurement structure to account for repeated measures at the same depth in the same year; the random effects included image location (depth), nested within plot, nested within ring. The model was run within R 3.6.3 [39] using the linear mixed model function (lme) within the nlme 3.1 package [40]. A similar approach was used for the PAW data, but the data were not transformed. The water use data over all depths (pre-flowering, post-flowering and season-long) were analyzed as a univariate model with [CO_2_] and cultivars as fixed factors and plot nested within ring in the random term. Graphs were produced with the ggplot2 3.3.0 package [41]. Significance was determined at α = 0.05, but α < 0.10 was considered marginally significant and discussed in the text.

## 5. Conclusions

Our results suggested soil water availability might play an important role in the stimulation of root growth under e[CO_2_]. In two dry seasons which had contrasting patterns of timing of water stress, root growth in deeper layers was indeed associated with increases in the yield response to e[CO_2_], likely through improved access to soil water later in the season, while increases in root growth in shallow layers were not. Presumably, it is more beneficial for the crop, in lentil at least, to allocate the additional carbon towards above-ground biomass and greater leaf area before flowering. Our results also suggested that for semi-arid areas, breeding for improved water extraction at depth, while beneficial in itself, might be particularly important to take advantage of the benefits of increasing e[CO_2_], provided there is sufficient plant available soil water at depth, and no intermediate dry soil layer to block root access to it.

## Figures and Tables

**Table 1:** Water use averaged over CO_2_ treatments in lentil in 2014 and 2015 in the Australian Grains Free Air CO_2_ Enrichment (AGFACE) facility.

Year	Cultivar	Pre-Flowering (mm)	Post-Flowering (mm)	Total (mm)
2014	PBA Ace	126 a ^1^	27 b	153 a
	*HS3010	97 b	49 a	146 a
2015	PBA Ace	100 A	99 A	198 A
	*HS3010	97 A	87 A	184 A

^1^ Data with the same letter in the same year are not statistically different from each other. *HS3010 is an abbreviation of line 05H010L-07HS3010.

## Data Availability

The data that support the findings of this study are available from the corresponding author (M.B.) upon reasonable request and approval from the former AGFACE program leaders (M.T. and G.J.F.).
